# Discovery of a Novel 4,5-Dihydro-1*H*-pyrazole-1-carbothioamide Derivative with Cytotoxic, Apoptotic, and ABL1 Inhibitory Activities Against Chronic Myeloid Leukemia

**DOI:** 10.3390/biomedicines14071651

**Published:** 2026-07-22

**Authors:** Ayben Erkan, Ayca Irgit Calayir, Halilibrahim Ciftci, Belgin Sever

**Affiliations:** 1 Department of Molecular Biology and Genetics, Burdur Mehmet Akif Ersoy University, Istiklal Campus, Burdur 15200, Türkiye; erkanayben7@gmail.com (A.E.); aycairgit@hotmail.com (A.I.C.); 2 Department of Biology, Burdur Mehmet Akif Ersoy University, Istiklal Campus, Burdur 15200, Türkiye; 3 Medicinal and Biological Chemistry Science Farm Joint Research Laboratory, Faculty of Life Sciences, Kumamoto University, Kumamoto 862-0973, Japan; 4 Department of Pharmaceutical Chemistry, Faculty of Pharmacy, Anadolu University, Eskisehir 26470, Türkiye

**Keywords:** chronic myeloid leukemia, ABL1 TKIs, drug discovery, chalcone, 4,5-dihydro-1*H*-pyrazole-1-carbothioamide

## Abstract

**Background/Objectives**: Chronic myeloid leukemia (CML) is a hematological malignancy driven by the constitutively active BCR-ABL1 fusion protein. Although current ABL1 tyrosine kinase inhibitors (TKIs) have improved CML management, resistance remains a major challenge, highlighting the need for novel therapeutic strategies. **Methods**: A chalcone derivative containing naphthalene and toluene moieties (**A**) was synthesized from 2-acetonaphthone and p-tolualdehyde and subsequently converted into a novel 4,5-dihydro-1*H*-pyrazole-1-carbothioamide derivative (**B**) through reaction with thiosemicarbazide. The cytotoxicity of compounds **A** and **B** against K562 CML cells was evaluated using the MTT assay. The active compound was further investigated for cytotoxic selectivity using HL-60 acute myeloid leukemia (AML) cells and healthy PBMCs. Apoptotic effects in K562 cells were analyzed using Annexin V/ethidium homodimer staining, whereas ABL1 inhibitory activity was determined using the ADP-Glo kinase assay. The potential interaction between the active compound and ABL1 was assessed by molecular docking analysis. **Results**: Compound **B** displayed potent cytotoxic activity against K562 cells with an IC_50_ value of 6.92 ± 1.14 µM and demonstrated selectivity toward leukemic cells over PBMCs (SI = 5.4). Treatment with compound **B** markedly induced apoptosis in K562 cells. In addition, compound **B** inhibited ABL1 activity in a concentration-dependent manner. Molecular docking studies revealed a favorable binding orientation within the ATP-binding pocket of ABL1. Furthermore, in silico absorption, distribution, metabolism, and excretion (ADME) analysis predicted favorable pharmacokinetic properties for compound **B**. **Conclusions:** These findings demonstrate that compound **B** possesses cytotoxic, pro-apoptotic, and ABL1 inhibitory activities against CML cells and may serve as a promising lead structure for the development of novel therapeutic agents targeting CML.

## 1. Introduction

Despite the remarkable clinical success of tyrosine kinase inhibitors (TKIs), chronic myeloid leukemia (CML) remains an important therapeutic challenge due to the emergence of drug resistance and disease progression. Consequently, the development of structurally novel anti-leukemic agents capable of overcoming these limitations remains an active area of medicinal chemistry research.

Hematological malignancies comprise leukemia, lymphoma, and multiple myeloma, arising from abnormal differentiation of hematopoietic stem cells [1,2,3,4]. Among these diseases, leukemia represents the leading cause of mortality and ranks among the ten most common causes of cancer-related death worldwide according to GLOBOCAN 2022 [5]. CML accounts for approximately 15% of newly diagnosed leukemia cases and is characterized by the presence of the Philadelphia chromosome, resulting from the reciprocal translocation t(9;22)(q34;q11) between the *ABL1* and *BCR* genes [6,7,8,9,10,11,12,13,14,15]. This translocation generates the constitutively active BCR-ABL1 fusion kinase, which continuously stimulates downstream signaling pathways, including MAPK, PI3K/AKT, and JAK/STAT, thereby promoting uncontrolled proliferation and suppressing apoptosis [13,14,15,16,17,18,19,20,21,22,23]. CML typically presents in a chronic phase but may progress to an aggressive blast phase if effective treatment is not achieved [24,25,26,27].

Targeting the constitutively active ABL1 kinase has transformed CML therapy. Six TKIs have been approved by the U.S. Food and Drug Administration (FDA), including the ATP-competitive inhibitors imatinib, dasatinib, nilotinib, bosutinib, and ponatinib, together with the allosteric inhibitor asciminib. Although these agents have dramatically improved patient survival, resistance mutations, inadequate response, and intolerance continue to limit long-term therapeutic success, highlighting the need for new molecular scaffolds with improved pharmacological profiles [26,27,28,29,30,31,32,33,34,35,36].

**Figure 1:**
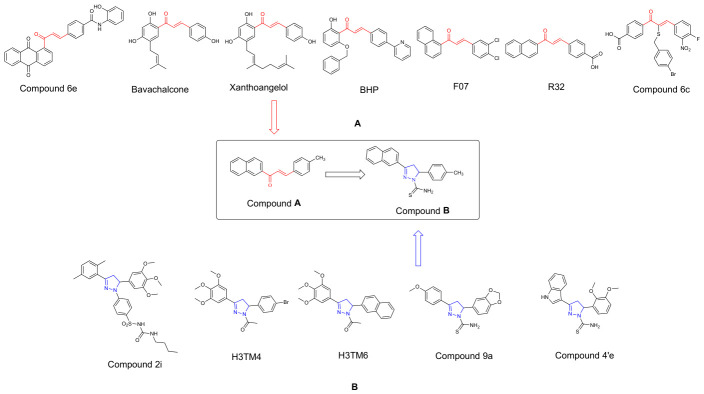
Representative chalcone- and pyrazoline-based anti-leukemic compounds reported in the literature, together with the molecular design of the synthesized compounds. Upper panel (**A**): representative chalcone derivatives with anti-leukemic activity. Lower panel (**B**): representative pyrazoline derivatives with anti-leukemic activity. The boxed area illustrates the molecular design strategy employed in this study, showing the conversion of the precursor chalcone (compound **A**) into the newly synthesized pyrazoline-1-carbothioamide derivative (compound **B**).

Among the numerous scaffolds investigated for anti-leukemic drug discovery, chalcones have attracted considerable attention because of their simple synthesis, structural diversity, and broad spectrum of biological activities, including anticancer, anti-inflammatory, antimicrobial, antiviral, antioxidant, and antidiabetic effects [37,38,39,40,41,42]. Their α,β-unsaturated carbonyl system functions as a Michael acceptor and may undergo covalent interactions with nucleophilic amino acid residues in target proteins while also contributing to modulation of multiple signaling pathways. Numerous chalcone derivatives have demonstrated potent activity against K562 CML cells, generally exhibiting low micromolar cytotoxicity. Representative examples include anthraquinone–chalcone hybrid 6e (IC_50_ = 3.87 μM), the prenylated chalcones bavachalcone (IC_50_ = 2.7 μM) and xanthoangelol (IC_50_ = 3.98 μM), BHP (IC_50_ = 4.1 μM), the 1-naphthylacetophenone derivative F07 (IC_50_ = 1.03 μM), R32 (IC_50_ = 9.47 μM), and the α-benzylthio chalcone 6c, which displayed both potent cytotoxicity against K562 cells (IC_50_ = 0.3 μM) and strong ABL1 inhibitory activity (IC_50_ = 0.5 μM) (Figure 1) [43,44,45,46,47,48,49]. Collectively, these studies establish chalcones as valuable starting points for the development of novel anti-CML agents.

Chalcones also serve as versatile synthetic intermediates for constructing biologically active heterocyclic systems, particularly pyrazolines. Among these, 2-pyrazoline derivatives have emerged as privileged scaffolds owing to their remarkable cytotoxic, antiproliferative, antioxidant, and kinase inhibitory properties [50,51,52,53,54,55]. Several pyrazoline derivatives have demonstrated promising anti-leukemic activity, including compounds 2i, H3TM4, H3TM6, 9a, and 4′e, which exhibited significant antiproliferative effects against leukemia cell lines, including K562 cells (Figure 1) [56,57,58,59]. These findings further support the therapeutic potential of pyrazoline-containing molecules in anti-leukemic drug discovery.

Encouraged by the well-documented anti-leukemic activities of both chalcone and pyrazoline scaffolds, we designed and synthesized a novel pyrazoline-1-carbothioamide derivative derived from a chalcone precursor. Although pyrazoline and carbothioamide moieties have individually been associated with diverse biological activities, including anticancer and kinase inhibitory effects, further investigation of pyrazoline-1-carbothioamide hybrids as anti-leukemic agents is warranted. Therefore, the synthesized compound was evaluated for its cytotoxic, apoptotic, and ABL1 kinase inhibitory activities against K562 CML cells.

Based on this rationale, we designed and synthesized a novel pyrazoline-1-carbothioamide derivative (compound **B**) (Figure 1) derived from a chalcone precursor (compound **A**) (Figure 1). The synthesized compounds were initially screened for cytotoxicity against K562 CML cells using the MTT assay. The active compound was subsequently evaluated for selectivity in HL-60 acute myeloid leukemia (AML) cells and peripheral blood mononuclear cells (PBMCs), while apoptosis induction was assessed by Annexin V/ethidium homodimer staining. Furthermore, ABL1 kinase inhibition was determined using the ADP-Glo kinase assay, and molecular docking studies were performed to investigate the potential binding interactions with the ABL1 kinase domain.

## 2. Materials and Methods

### 2.1. Chemistry

All chemicals were obtained from commercial suppliers and used as supplied. Melting points were determined with a MP90 instrument (Mettler Toledo, Columbus, OH, USA). Thin-layer chromatography on silica gel 60 F254 plates (Merck, Darmstadt, Germany) was employed to follow reaction progress and evaluate product purity. Structural characterization was performed using ^1^H and ^13^C NMR spectroscopy on a Bruker instrument (Billerica, MA, USA), while accurate mass measurements were obtained with a JEOL JMS-700 Station/JMS-BU-20-GCmate system (Akishima, Tokyo, Japan).

#### 2.1.1. Synthesis of (E)-1-(Naphthalen-2-yl)-3-(p-tolyl)prop-2-en-1-one (**A**)

A mixture of 2-acetonaphthone (1 mmol) and p-tolualdehyde (1 mmol) was treated with NaOH (1.1 mmol) in ethanol and stirred at room temperature for 24 h. Reaction progress was followed by thin-layer chromatography (TLC). After completion, the reaction medium was transferred onto crushed ice, inducing precipitation of the desired chalcone. The precipitated material was separated by filtration, washed with distilled water, and allowed to dry. Final purification was carried out by recrystallization from ethanol, yielding compound **A** in high purity [60,61,62].

Compound **A** [63,64]: pale yellow powder. Yield: 92%. M.p. 93–94 °C. ^1^H NMR (500 MHz, *CDCl*_3_) *δ* (ppm): 2.37 (3H, s, CH_3_), 7.22 (2H, d, *J* = 9.5 Hz), 7.52–7.59 (4H, m), 7.63 (1H, d, *J* = 15.6 Hz), 7.85 (1H, d, *J* = 16.0 Hz), 7.88 (1H, s), 7.91 (1H, d, *J* = 9.2 Hz), 7.97 (1H, d, *J* = 8.0 Hz), 8.09 (1H, d, *J* = 8.6 Hz), 8.52 (s, 1H). ^13^C NMR (125 MHz, *CDCl*_3_) *δ* (ppm): 21.5 (CH_3_), 121.2 (CH), 124.5 (CH), 126.8 (CH), 127.9 (CH), 128.4 (CH), 128.6 (2CH), 129.5 (CH), 129.8 (3CH), 129.9 (CH), 132.2 (C), 132.6 (C), 135.5 (C), 135.8 (C), 141.2 (C), 144.9 (CH), 190.4 (C, C=O). HRMS (FAB) calcd. for C_20_H_16_O [M+H]^+^: m/z = 273.1279; found: 273.1280. (Spectral Data: Supplementary Materials, Figures S1–S3).

#### 2.1.2. Synthesis of 3-(Naphthalen-2-yl)-5-(p-tolyl)-4,5-dihydro-1*H*-pyrazole-1-carbothioamide (**B**)

To a solution of compound **A** (3 mmol) in ethanol, thiosemicarbazide (4.5 mmol) and NaOH (3 mmol) were added. The reaction mixture was heated under reflux and maintained under these conditions for 8–12 h, while the conversion was monitored by TLC. After completion, the mixture was allowed to reach room temperature and then transferred into ice-cold water. The resulting solid was separated by vacuum filtration, rinsed with water to remove residual impurities, and dried. Final purification was performed by recrystallization from ethanol, yielding compound **B** [60,61,62].

Compound **B**: yellow crystalline powder. Yield: 80%. M.p. 184–185 °C. ^1^H NMR (500 MHz, *CDCl*_3_) *δ* (ppm): *δ* (ppm): 2.29 (3H, s), 3.31 (1H, dd, *J_AB_* = 17.2 Hz, *J_AX_* = 3.9 Hz), 3.89 (1H, dd, *J_BA_* = 17.7 Hz, *J_BX_* = 11.8 Hz), 6.05 (1H, dd, *J_BX_* = 10.9 Hz, *J_AX_* = 3.4 Hz), 7.14 (4H, s), 7.49–7.55 (2H, m), 7.81–7.86 (3H, m), 7.93 (1H, s), 7.99 (1H, d, *J* = 8.9 Hz). ^13^C NMR (125 MHz, *CDCl*_3_) *δ* (ppm): 21.0 (CH_3_), 43.1 (CH_2_), 63.4 (CH), 123.4 (CH), 125.4 (2CH), 126.9 (CH), 127.7 (CH), 127.9 (CH), 128.1 (CH), 128.3 (CH), 128.5 (CH), 128.7 (C), 129.6 (2CH), 132.9 (C), 134.4 (C), 137.3 (C), 138.9 (C), 156.0 (C), 176.8 (C). HRMS (FAB) calcd. for C_21_H_19_N_3_S [M+H]^+^: m/z = 346.1378; found: 346.1405. (Spectral Data: Supplementary Materials, Figures S4–S6).

### 2.2. Biological Activity

#### 2.2.1. Cytotoxicity

K562 cells were selected because they express the constitutively active BCR-ABL1 fusion kinase and therefore represent the most widely used in vitro model of CML for evaluation of ABL1-targeted compounds. HL-60 cells do not express the BCR-ABL1 fusion protein and were therefore selected as a leukemia cell line lacking constitutive ABL1 activation, enabling preliminary assessment of target-related selectivity. K562 and HL-60 cell lines (ATCC, Manassas, VA, USA) and peripheral blood mononuclear cells (PBMCs) were cultured in RPMI 1640 medium (Wako Pure Chemical Industries, Osaka, Japan) supplemented with 10% fetal bovine serum (FBS) (Biosera, Kansas City, MO, USA). PBMCs were seeded in 96-well plates at 1 × 10^6^ cells/mL and incubated for 72 h. K562 and HL-60 cells were plated at a final density of 4 × 10^4^ cells/mL in 24-well plates and incubated for 72 h. For initial cytotoxicity screening, K562 cells were treated with the synthesized compounds A and B at 10 µM, along with imatinib as a reference. Cells were incubated for 48 h. The MTT assay (Dojindo Molecular Technologies, Kumamoto, Japan) was performed as previously described [65,66,67,68,69]. A total of 100 µL of MTT solution was added to each well, followed by incubation for 4 h. After incubation, the culture medium was removed, and the formed formazan crystals were solubilized in 100 µL of DMSO. Absorbance was measured at 570 nm using an Infinite M1000 plate reader (Tecan, Männedorf, Switzerland). Experiments were performed in triplicate. Based on the initial cytotoxicity screening results, the active compound was further evaluated for its selectivity in leukemic cell lines K562 and HL-60 compared with PBMCs using the MTT assay in a dose-dependent manner. IC_50_ values for each cell line were calculated as the concentration of the compound that reduced absorbance by 50% relative to the control [65,66,67,68,69].

#### 2.2.2. Apoptosis Assay

K562 cells were seeded in 24-well plates at 4 × 10^4^ cells/well and treated with compound B at its defined IC_50_ concentration, followed by 12 h and 24h of incubation. Apoptotic, necrotic, and viable cells were assessed using an apoptotic/necrotic/healthy cell detection kit (PromoKine, Heidelberg, Germany), according to the manufacturer’s instructions with slight modifications. Cells were washed twice with 1× binding buffer and incubated in a staining solution containing 50 µL of 1× binding buffer and 4 µL each of FITC-Annexin V, ethidium homodimer III, and Hoechst 33,342 for 30 min at room temperature in the dark. After staining, apoptotic, late apoptotic, and necrotic cells were visualized using a Biorevo BZ-9000 fluorescence microscope (Keyence, Osaka, Japan). Annexin V-positive/ethidium homodimer-negative cells were considered early apoptotic, whereas Annexin V-positive/ethidium homodimer-positive cells were classified as late apoptotic due to membrane permeabilization occurring at later stages of apoptosis [66,67].

#### 2.2.3. ABL1 Inhibition Assay

The ABL1 inhibitory activity was analyzed using the ABL1 enzyme assay (Promega V1901, Promega Corporation, Madison, WI, USA) according to the manufacturer’s protocol. Kinase reactions were prepared in 384-well plates by combining 4 µL of kinase solution, 4 µL of ATP/substrate mixture, and 2 µL of the active compound at concentrations of 100, 10, 1, and 0.1 μM, or 5% DMSO as a control. The plates were incubated at room temperature for 1.5 h, and kinase activity was measured using the ADP-Glo Kinase Assay (Promega Corporation, Madison, WI, USA). Briefly, 5 µL of ADP-Glo reagent was added to each well and incubated at room temperature for 40 min to terminate the kinase reaction. Subsequently, 10 µL of Kinase Detection Reagent was added, and the plate was incubated for an additional 30 min to allow conversion of ADP to ATP and generation of a luminescent signal. The inhibitory activity against ABL1 kinase was quantified in a dose-dependent manner using an Infinite M1000 luminescence plate reader (Tecan, Grödig, Austria) [66,67].

### 2.3. In Silico Studies

The X-ray structure of ABL (PDB ID: 2HYY) was downloaded from the Protein Data Bank [70]. Protein and ligand preparation procedures were carried out in Maestro [71], followed by geometry optimization using the OPLS force field. Docking calculations for compound B and imatinib were performed with Glide in SP mode to explore their binding preferences within the ATP-binding cavity of ABL1 TK. The generated complexes were analyzed in terms of binding orientation and intermolecular interactions. Drug-likeness, pharmacokinetic parameters, and toxicity predictions were further assessed using SwissADME [72].

## 3. Results

### 3.1. Chemistry

**Scheme 1:**
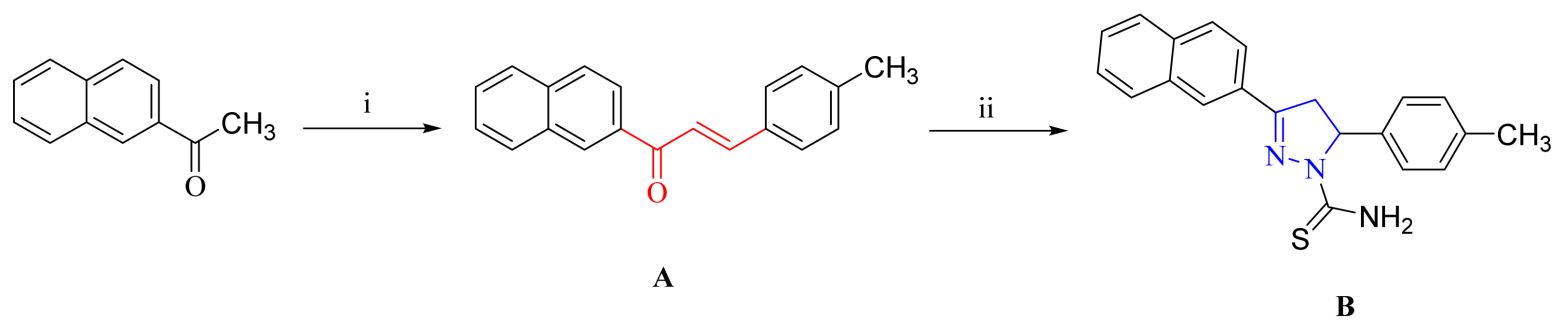
Synthesis of compounds **A** and **B**: (i) p-tolualdehyde, NaOH, ethanol, rt, 24 h; (ii) Thiosemicarbazide, NaOH, ethanol, reflux, 8–12 h.

Compound **A** was prepared from 2-acetonaphthone and p-tolualdehyde through a base-mediated condensation reaction, providing the desired chalcone in excellent yield (92%). Subsequent treatment of this intermediate with thiosemicarbazide under alkaline conditions afforded compound **B**, which was also obtained in high yield (80%) (Scheme 1).

**Figure 2:**
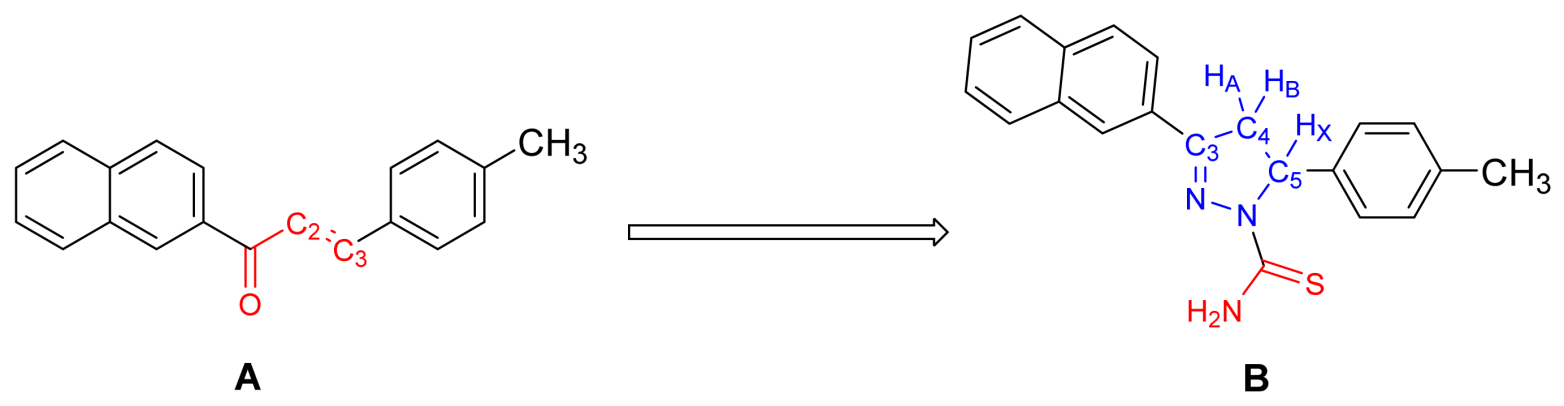
NMR diagrams for compounds **A** and **B**.

The structures of compounds **A** and **B** were elucidated through a combination of spectroscopic techniques, including ^1^H nuclear magnetic resonance (NMR), ^13^C NMR, and high-resolution mass spectrometry (HRMS). For compound **A**, the ^1^H NMR spectrum exhibited two characteristic doublets at δ 7.63 (*J* = 15.6 Hz) and δ 7.85 (*J* = 16.0 Hz), corresponding to the olefinic protons of the propenone fragment. The large coupling constants are consistent with a trans (*E*)-configured α,β-unsaturated carbonyl system. The spectrum also displayed the expected aromatic proton resonances. In the ^13^C NMR spectrum, the carbon atoms of the propenone moiety were identified at δ 121.2 (C_2_), δ 144.9 (C_3_), and δ 190.4 ppm (carbonyl carbon), supporting the proposed chalcone structure (Figure 2). The structural assignment of compound **B** was confirmed by the formation of the pyrazoline ring, as evidenced by the characteristic ABX spin system observed in the ^1^H NMR spectrum. The ring H_A_, H_B_, and H_X_ protons appeared as doublets of doublets at δ 3.31 (*J_AB_* = 17.2 Hz, *J_AX_* = 3.9 Hz), δ 3.89 (*J_BA_* = 17.7 Hz, *J_BX_* = 11.8 Hz), and δ 6.05 ppm (*J_AX_* = 3.4 Hz, *J_BX_* = 10.9 Hz), respectively. These signals are characteristic of pyrazoline derivatives and confirm successful cyclization. Consistent with this assignment, the ^13^C NMR spectrum showed resonances at δ 156.0, 43.1, and 63.4 ppm, attributable to the C_3_, C_4_, and C_5_ carbons of the pyrazoline ring, respectively. Furthermore, the signal observed at δ 176.8 ppm was assigned to the thiocarbonyl (C=S) carbon, providing additional evidence for the proposed structure. The absence of detectable NH proton signals in the ^1^H NMR spectrum is likely due to rapid proton exchange processes and/or solvent-related effects (Figure 2).

### 3.2. Anticancer Activity

**Figure 3:**
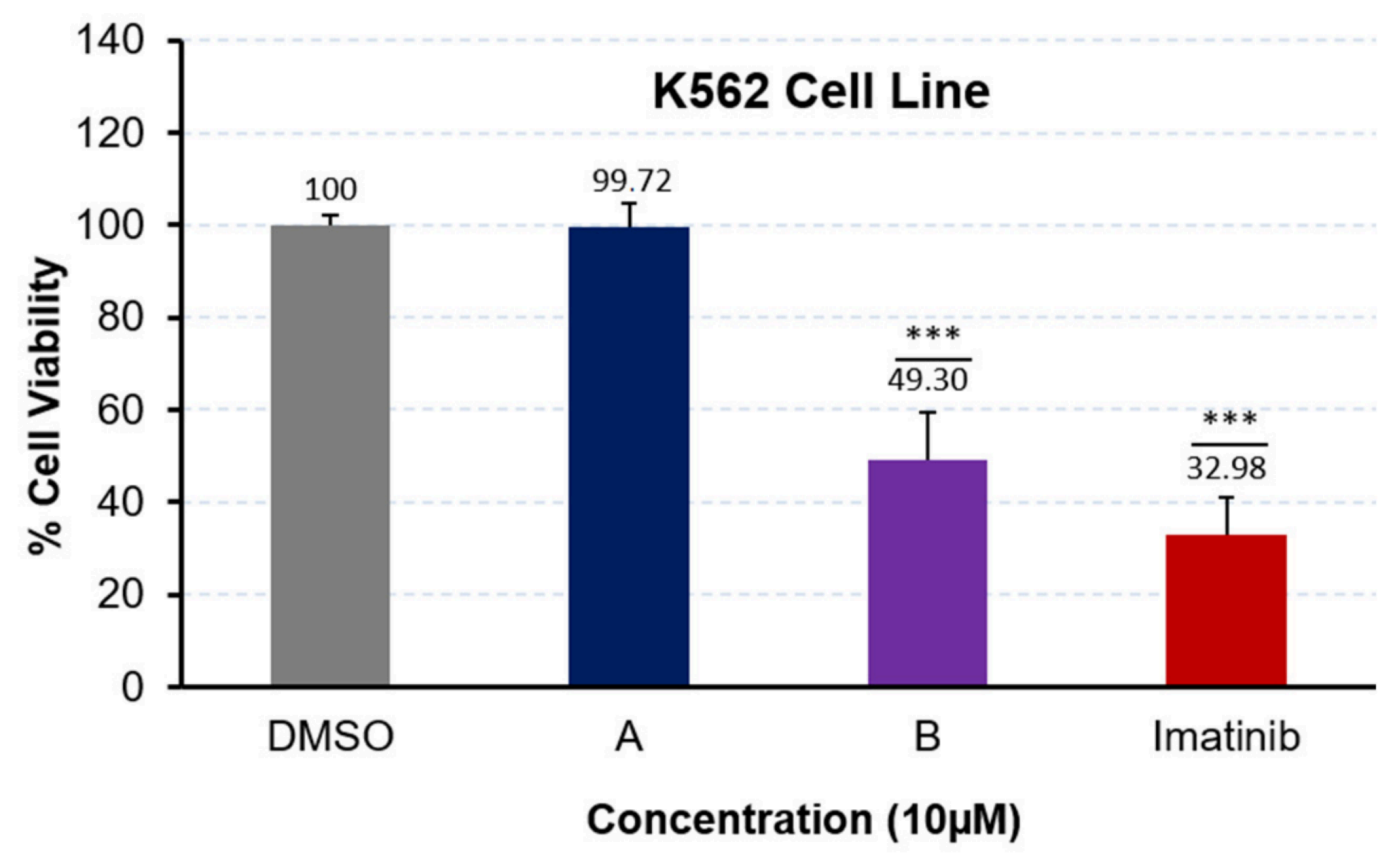
Initial cytotoxicity screening of the synthesized compounds **A** and **B** compared with imatinib as a reference at a 10 µM concentration against K562 cells after 48 h incubation. Data are presented as mean ± SD from three independent experiments; DMSO was set as 100% viability; *** *p* < 0.001.

The synthesized compounds **A** and **B** were evaluated for cytotoxicity against K562 cells using the MTT assay. In the initial cytotoxicity screening (Figure 3), compound **A** showed no detectable cytotoxic activity at a 10 µM concentration, whereas compound **B** reduced K562 cell viability by approximately 51%.

**Figure 4:**
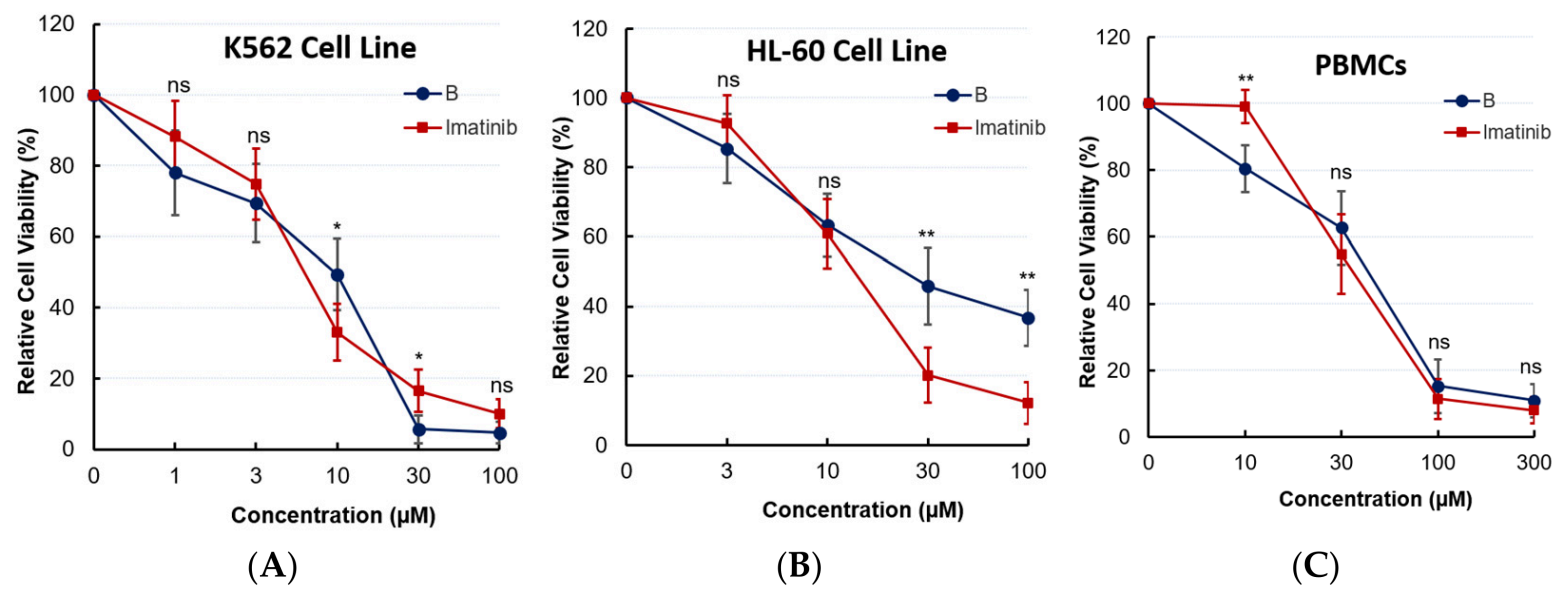
Dose-dependent cytotoxicity of compound **B** in K562 cells (**A**), HL-60 cells (**B**), and PBMCs (**C**), using imatinib as the reference. Data are presented as mean ± SD (*n* = 3); * *p* < 0.05, ** *p* < 0.01, ns: not significant.

Compound **B** was further evaluated for its concentration-dependent cytotoxic activity against K562 cells (Figure 4A). Treatment with compound **B** resulted in a gradual decrease in cell viability as the concentration increased. At concentrations of 30 μM and 100 μM, cell viability was reduced to below 10%, indicating a pronounced cytotoxic effect. The activity profile of compound **B** was comparable to that of imatinib throughout the tested concentration range. Consistent with these observations, compound **B** exhibited potent anti-leukemic activity against K562 cells, with an IC_50_ value of 6.92 ± 1.14 μM, which was close to that of imatinib (IC_50_ = 6.07 ± 1.32 μM) (Table 1).

The cytotoxic effect of compound **B** was also evaluated in HL-60 cells (Figure 4B). Both compound **B** and imatinib produced a concentration-dependent reduction in cell viability. At lower concentrations (3–10 μM), the two compounds displayed comparable activity. However, at 30 and 100 μM, imatinib exerted a more pronounced cytotoxic effect, whereas a substantial fraction of HL-60 cells remained viable following compound B treatment. Accordingly, compound **B** exhibited a higher IC_50_ value (21.50 ± 2.53 μM) than imatinib (12.55 ± 1.82 μM), indicating lower sensitivity of HL-60 cells to compound **B** (Table 1). The preferential activity of compound B toward K562 cells compared with HL-60 cells may be associated, at least in part, with its inhibitory effect on ABL1 kinase.

The cytotoxic effects of compound B on non-malignant PBMCs were also examined to assess its selectivity (Figure 4C). Compound **B** produced a concentration-dependent reduction in PBMC viability, with a response pattern generally comparable to that observed for imatinib. While compound **B** caused a slightly greater decrease in cell viability at lower concentrations, both compounds exhibited marked cytotoxicity at 100 and 300 μM. The IC_50_ value of compound **B** against PBMCs was determined as 37.23 ± 4.36 μM, which was comparable to that of imatinib (32.02 ± 4.47 μM) (Table 1).

**Figure 5:**
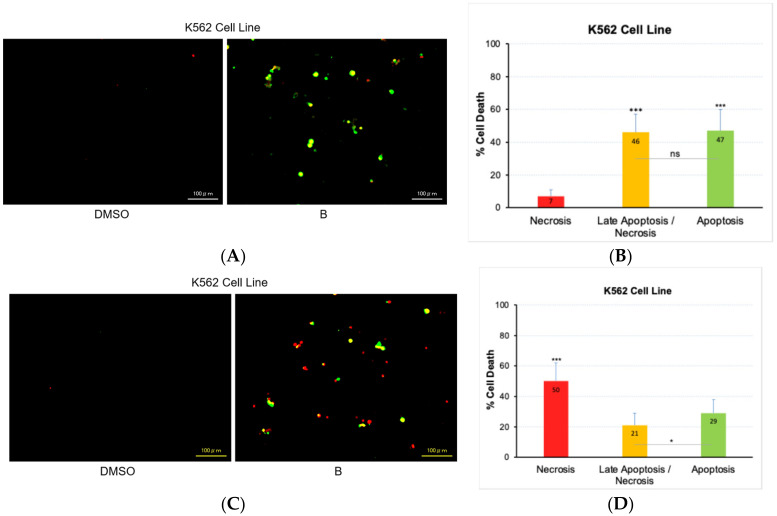
Time-dependent apoptosis induced by compound **B** in K562 cells. Representative fluorescence images of DMSO-treated control cells and cells treated with compound **B** at its IC_50_ concentration for 12 h (**A**) and 24 h (**C**). Annexin V-positive/ethidium homodimer-negative cells were classified as early apoptotic, whereas Annexin V-positive/ethidium homodimer-positive cells were classified as late apoptotic. Viable and early apoptotic cells are shown in green, whereas ethidium homodimer-positive cells (late apoptotic/necrotic) are shown in red. Scale bar = 100 μm. Quantitative analysis of cell death after 12 h (**B**) and 24 h (**D**) treatment. Data are presented as mean ± SD (*n* = 3). * *p* < 0.05, *** *p* < 0.001, ns: not significant.

The ability of compound B to induce apoptosis in K562 cells was investigated using Annexin V/Ethidium Homodimer staining after 12 h and 24 h of treatment (Figure 5). As shown in Figure 5A,B, compound **B** markedly promoted apoptotic cell death after 12 h, with 47% and 46% of the cell population detected in the early and late apoptotic stages, respectively, whereas only 7% of the cells were classified as necrotic. These findings indicate that compound B primarily induces programmed cell death through apoptotic mechanisms rather than nonspecific necrotic damage. However, prolonging the incubation period to 24 h (Figure 5C,D) resulted in an increased proportion of necrotic cells (50%), accompanied by a reduction in the percentages of early and late apoptotic cells to 29% and 21%, respectively. This shift may reflect the progression of apoptotic cells to secondary necrosis following prolonged exposure to compound **B**.

**Figure 6:**
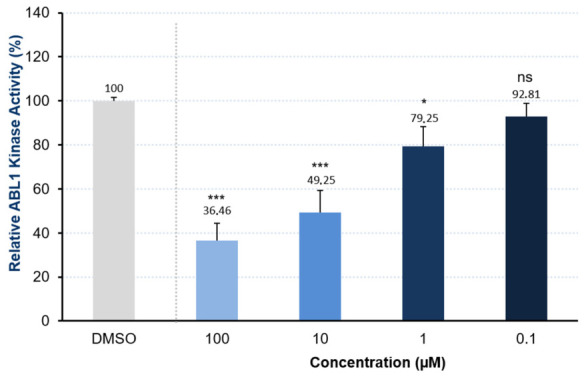
Effect of compound **B** on ABL1 activity determined by the ADP-Glo kinase assay. Relative ABL1 inhibitory activity was expressed as a percentage of the DMSO-treated control, which was set to 100%. Compound **B** reduced ABL1 activity in a concentration-dependent manner, with the strongest effect observed at 100 µM. Data are presented as mean ± SD from three independent experiments. * *p* < 0.05, *** *p* < 0.001, ns: not significant.

Since constitutive activation of ABL1 plays a central role in the pathogenesis and progression of CML, the effect of compound **B** on ABL1 activity was further investigated using the ADP-Glo kinase assay. Compound **B** reduced ABL1 activity in a concentration-dependent manner (Figure 6). Relative ABL1 activity decreased progressively with increasing compound concentration, reaching 49% and 36% of the control level at 10 and 100 μM, respectively. These values correspond to ABL1 inhibition rates of 51% and 64%. The strongest inhibitory effect was observed at 100 μM, indicating that compound **B** is capable of suppressing ABL1 activity at micromolar concentrations.

### 3.3. In Silico Studies

**Figure 7:**
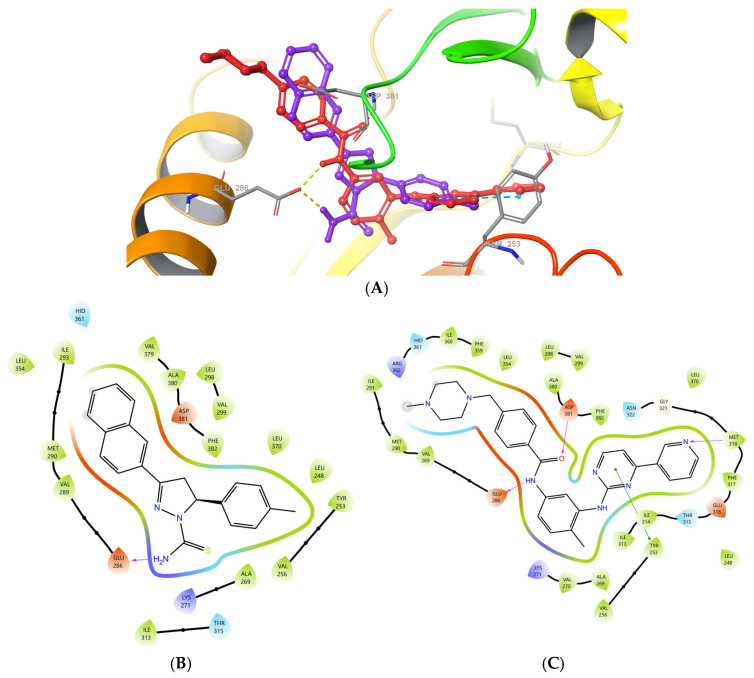
Overlay of the docked conformations of compound **B** (purple) and imatinib (red) within the catalytic pocket (**A**). Two-dimensional representation of the interactions formed between compound **B** and ABL1 residues (**B**). Two-dimensional interaction profile of imatinib within the ABL1 binding cavity (**C**). Hydrogen bonds and π–π interactions are depicted as purple arrows and green lines, respectively.

To explore the possible molecular basis of ABL1 inhibition, compound **B** was docked into the ATP-binding pocket of ABL1 (PDB ID: 2HYY) [70] through Maestro software [71] compared with the reference inhibitor imatinib (Figure 7A). Compound **B** was accommodated within the ATP-binding cleft with a docking score of -9.188 kcal/mol, whereas imatinib exhibited a more favorable binding energy of -12.370 kcal/mol. Docking analysis indicated that compound **B** forms a hydrogen-bond interaction with Glu286, while its aromatic framework is stabilized by hydrophobic contacts with surrounding residues, including Val289, Met290, Ile293, Leu298, Val299, Ala380, and Phe382 (Figure 7B). On the other hand, imatinib established a more extensive interaction network involving key residues such as Glu286, Asp381, Met318, and Tyr253, which are known to contribute to high-affinity ABL1 binding (Figure 7C). The absence of these additional interactions in compound **B** may account for its lower predicted binding affinity compared with imatinib. Nevertheless, the ability of compound **B** to occupy the ATP-binding pocket and interact with Glu286 is consistent with its experimentally observed ABL1 inhibitory activity.

**Figure 8:**
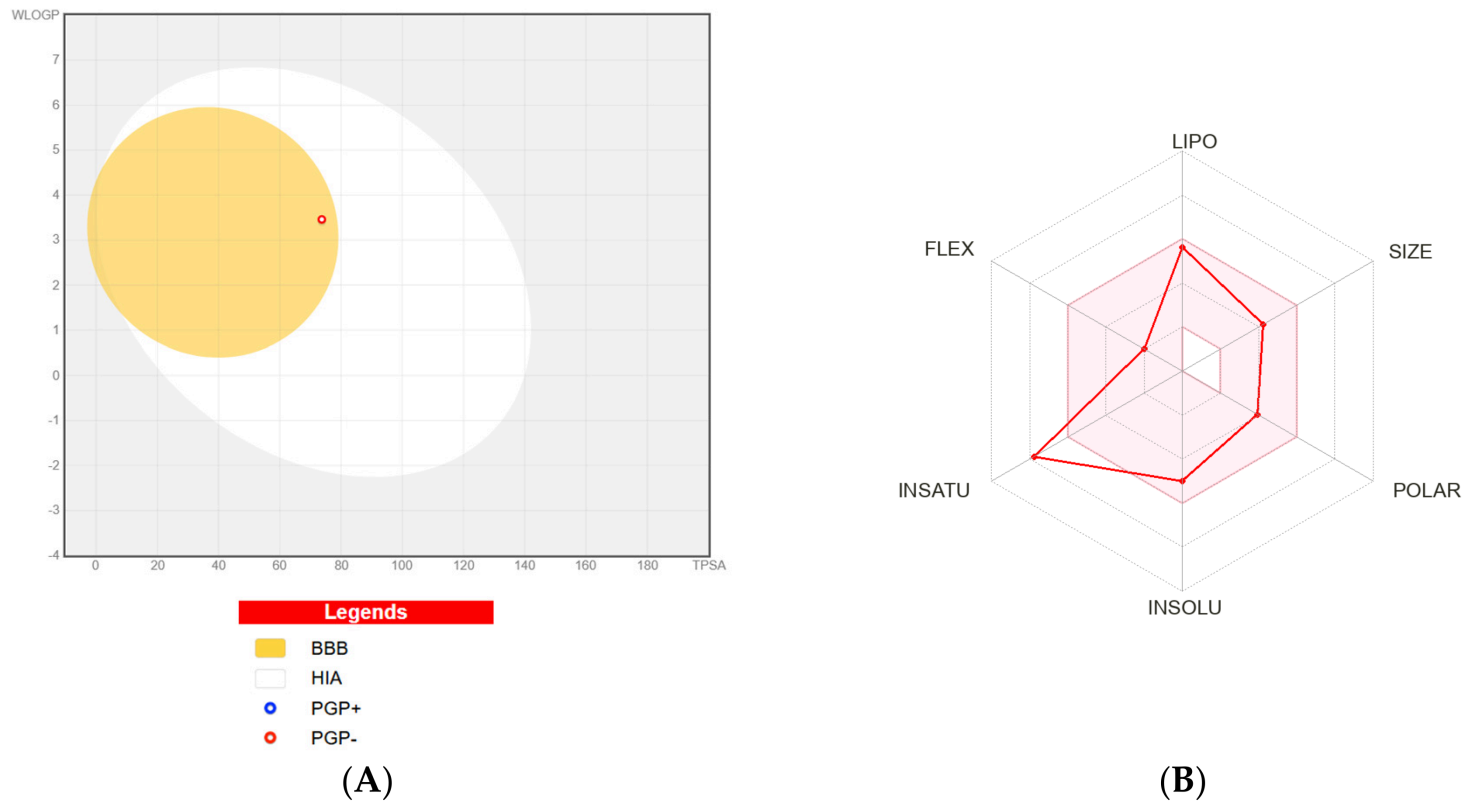
The BOILED-Egg model (**A**) and the bioavailability radar (**B**) of compound B.

The SwissADME analysis [72] indicated that compound **B** possesses favorable physicochemical, pharmacokinetic, and drug-likeness properties. The compound exhibited a molecular weight of 345.46 g/mol, a topological polar surface area (TPSA) of 73.71 Å^2^, three rotatable bonds, one hydrogen bond acceptor, and one hydrogen bond donor. Its consensus LogP value of 4.19 suggests moderate lipophilicity. According to the BOILED-Egg model (Figure 8A), compound **B** is predicted to exhibit high gastrointestinal absorption and blood–brain barrier (BBB) permeability. Furthermore, the molecule was identified as a non-substrate of P-glycoprotein (P-gp), indicating a lower likelihood of active efflux. Drug-likeness evaluation demonstrated full compliance with Lipinski, Ghose, Veber, Egan, and Muegge criteria, with a calculated bioavailability score of 0.55. The bioavailability radar (Figure 8B) further revealed that most physicochemical parameters, including lipophilicity, size, polarity, flexibility, and solubility, fall within the optimal range for oral bioavailability. A minor deviation was observed in the unsaturation parameter, which may be attributed to the highly aromatic nature of the molecule. Additionally, no PAINS alerts were detected, while a single Brenk alert related to the thiocarbonyl group was identified.

## 4. Discussion

The present study describes the synthesis and biological evaluation of a novel pyrazoline-1-carbothioamide derivative bearing naphthalene and p-tolyl substituents as a potential anti-leukemic agent. The molecular design was inspired by the well-established anticancer activities of both chalcone and pyrazoline scaffolds. Chalcones have attracted considerable attention as anti-leukemic agents, while pyrazoline derivatives have emerged as privileged structures in medicinal chemistry due to their diverse biological activities, including antiproliferative and kinase inhibitory effects.

One of the most notable findings of this study was the marked enhancement in biological activity following transformation of the chalcone intermediate (compound **A**) into the corresponding pyrazoline-1-carbothioamide derivative (compound **B**). While compound **A** did not exhibit measurable cytotoxicity against K562 cells at 10 μM, compound B reduced cell viability by approximately 51% at the same concentration. This observation suggests that cyclization of the α,β-unsaturated carbonyl system into a pyrazoline ring, together with incorporation of a carbothioamide moiety, substantially improves anti-leukemic activity. The increased activity may be associated with the greater conformational rigidity of the pyrazoline scaffold and the introduction of additional heteroatoms capable of participating in favorable intermolecular interactions with biological targets.

The design of compound **B** was guided by structural features commonly observed in biologically active pyrazoline derivatives. Several anti-leukemic pyrazolines reported in the literature possess extended aromatic systems, including naphthalene, indole, and benzodioxole moieties, which are thought to promote favorable hydrophobic and π-mediated interactions with biological targets. In particular, the naphthalene-containing derivative H3TM6 and the indole-based derivative 4′e demonstrated promising activity against K562 cells. Based on these observations, a naphthalene ring was incorporated into compound **B** to provide an expanded aromatic surface, while a p-tolyl substituent was introduced to further enhance hydrophobic character and maintain synthetic accessibility. In addition, compound **B** contains a carbothioamide functionality, a structural feature also presents in compounds 9a and 4′e. The presence of this group may contribute to biological activity through additional hydrogen-bonding interactions and improved target recognition. Notably, compound **B** exhibited an IC_50_ value of 6.92 μM against K562 cells, demonstrating greater activity than H3TM4 and H3TM6 and remaining within the activity range reported for promising pyrazoline-based anti-leukemic agents. These findings suggest that the combination of a pyrazoline nucleus with naphthalene and carbothioamide pharmacophoric elements represents a favorable structural framework for the development of new anti-CML compounds. An additional noteworthy observation was the preferential activity of compound B toward K562 cells compared with HL-60 cells. The compound was approximately threefold less active against HL-60 cells and displayed an SI of 5.4 relative to PBMCs. Because K562 cells are characterized by constitutive BCR-ABL1 signaling, whereas HL-60 cells are not dependent on this pathway, the observed preferential activity toward K562 cells may be partially associated with inhibition of ABL1 activity. However, the moderate level of ABL1 inhibition suggests that additional molecular targets or signaling pathways may also contribute to the overall cytotoxic effect of compound **B**. Therefore, ABL1 inhibition is likely one of several mechanisms underlying the observed anti-leukemic activity rather than the sole determinant of its biological effects.

Molecular docking studies provided additional insight into the potential mechanism underlying ABL1 inhibition. Compound **B** was successfully accommodated within the ATP-binding pocket of ABL1 TK and formed a hydrogen-bond interaction with Glu286. Although its docking score was lower than that of imatinib, the predicted binding mode supports the experimental kinase inhibition data. The weaker binding affinity relative to imatinib may be explained by the absence of interactions with key residues such as Asp381, Met318, and Tyr253, which are known to contribute significantly to high-affinity ABL1 binding. Nevertheless, the ability of compound **B** to occupy the ATP-binding cleft and interact with catalytically relevant residues suggests that the pyrazoline-1-carbothioamide scaffold may serve as a useful platform for future optimization. Although the observed ABL1 inhibition was lower than that of clinically used inhibitors, these findings indicate that the scaffold provides a promising starting point for further structural optimization to improve kinase potency and selectivity.

In addition to its biological activity, compound **B** exhibited a favorable predicted pharmacokinetic profile. The compound complied with the major drug-likeness criteria, including the Lipinski, Ghose, Veber, Egan, and Muegge rules, and was predicted to possess high gastrointestinal absorption. Furthermore, no PAINS alerts were detected, supporting the suitability of the scaffold for further medicinal chemistry studies. Although a Brenk alert associated with the thiocarbonyl functionality was identified, the overall ADME profile suggests that compound **B** possesses physicochemical characteristics compatible with lead-like molecules and warrants further investigation.

Overall, the present findings demonstrate that conversion of a naphthalene-containing chalcone into a pyrazoline-1-carbothioamide derivative generated a molecule with enhanced anti-leukemic activity, the ability to induce apoptosis, measurable ABL1 inhibition, and favorable predicted pharmacokinetic properties. These results highlight the potential of this scaffold as a promising starting point for the development of novel therapeutic agents targeting CML.

## 5. Conclusions

A chalcone precursor (compound **A**) and a novel pyrazoline-1-carbothioamide derivative (compound **B**) were synthesized and evaluated for their anti-leukemic potential. Compound **B** displayed significant cytotoxic and pro-apoptotic effects against K562 cells and showed inhibitory activity toward ABL1. These results highlight the therapeutic potential of pyrazoline-1-carbothioamide derivatives and support further investigation of compound **B** as a candidate for CML treatment.

## Figures and Tables

**Table 1:** The IC_50_ values of compound B and imatinib against K562 and HL60 cells and PBMCs.

Compound	IC_50_ Values (μM)			SI *
	K562	HL-60	PBMCs	
**B**	6.92 ± 1.14	21.50 ± 2.53	37.23 ± 4.36	5.4
**Imatinib**	6.07 ± 1.32	12.55 ± 1.82	32.02 ± 4.47	6.1

* SI = Selectivity Index (IC_50_ for PBMC/IC_50_ for K562 cell line).

## Data Availability

The original contributions presented in this study are included in the article/Supplementary Materials. Further inquiries can be directed to the corresponding authors.

## Supplementary Materials

The following supporting information can be downloaded at https://www.mdpi.com/article/10.3390/biomedicines14071651/s1, Figure S1: ^1^H NMR Spectrum of compound **A**; Figure S2: ^13^C NMR Spectrum of compound **A**; Figure S3: Mass Spectrum of compound **A**; Figure S4: ^1^H NMR Spectrum of compound **B**; Figure S5: ^13^C NMR Spectrum of compound **B**; Figure S6: Mass Spectrum of compound **B**.

## Abbreviations

The following abbreviations are used in this manuscript:

ABL1	Abelson murine leukemia viral oncogene homolog 1
ADME	Absorption, distribution, metabolism, and excretion
AML	Acute myeloid leukemia
BBB	Blood–brain barrier
BCR	Breakpoint cluster region
CML	Chronic myeloid leukemia
FDA	U.S. Food and Drug Administration
JAK/STAT	Janus kinase/signal transducers and activators of transcription
HL-60	Human acute promyelocytic leukemia cell line
HRMS	High-resolution mass spectrometry
IC_50_	Half-maximal inhibitory concentration
K562	Human chronic myeloid leukemia cell line
MAPK	Mitogen-activated protein kinase
MTT	3-(4,5-Dimethylthiazol-2-yl)-2,5-diphenyltetrazolium bromide
NMR	Nuclear magnetic resonance
PBMCs	Peripheral blood mononuclear cells
PI3K/AKT	Phosphoinositide 3-kinase/protein kinase B
P-gp	P-glycoprotein
SD	Standard deviation
SI	Selectivity Index
TKIs	Tyrosine kinase inhibitors
TPSA	Topological polar surface area
