# Supplementary material for: Discovery of a Novel 4,5-Dihydro-1H-pyrazole-1-carbothioamide Derivative with Cytotoxic, Apoptotic, and ABL1 Inhibitory Activities Against Chronic Myeloid Leukemia
Source: Biomedicines. 2026 Jul 22;14(7):1651. doi: 10.3390/biomedicines14071651 (PMC13406097; doi:10.3390/biomedicines14071651)

Supplementary Information

Discovery of a Novel 4,5-Dihydro-1*H*-pyrazole-1-carbothioamide Derivative with Cytotoxic, Apoptotic, and ABL1 Inhibitory Activities Against Chronic Myeloid Leukemia

Ayben Erkan[1], Ayca Irgit Calayir[1,2], Halilibrahim Ciftci[1,3,*], Belgin Sever[1,4,*]

[1] Department of Molecular Biology and Genetics, Burdur Mehmet Akif Ersoy University, Istiklal Campus, Burdur 15200, Türkiye; erkanayben7@gmail.com; aycairgit@hotmail.com; hciftci@mehmetakif.edu.tr; belginsever@anadolu.edu.tr
[2] Department of Biology, Burdur Mehmet Akif Ersoy University, Istiklal Campus, Burdur 15200, Türkiye
[3] Medicinal and Biological Chemistry Science Farm Joint Research Laboratory, Faculty of Life Sciences, Kumamoto University, Kumamoto 862-0973, Japan
[4] Department of Pharmaceutical Chemistry, Faculty of Pharmacy, Anadolu University, Eskisehir 26470, Türkiye

*** Correspondence:** hciftci@mehmetakif.edu.tr and belginsever@anadolu.edu.tr

Supplementary Figures

Figure S1: ^{1}H NMR Spectrum of compound **A**
Figure S2: ^{13}C NMR Spectrum of compound **A**
Figure S3: Mass Spectrum of compound **A**
Figure S4: ^{1}H NMR Spectrum of compound **B**
Figure S5: ^{13}C NMR Spectrum of compound **B**
Figure S6: Mass Spectrum of compound **B**

Figure S1: ^{1}H NMR Spectrum of compound **A**

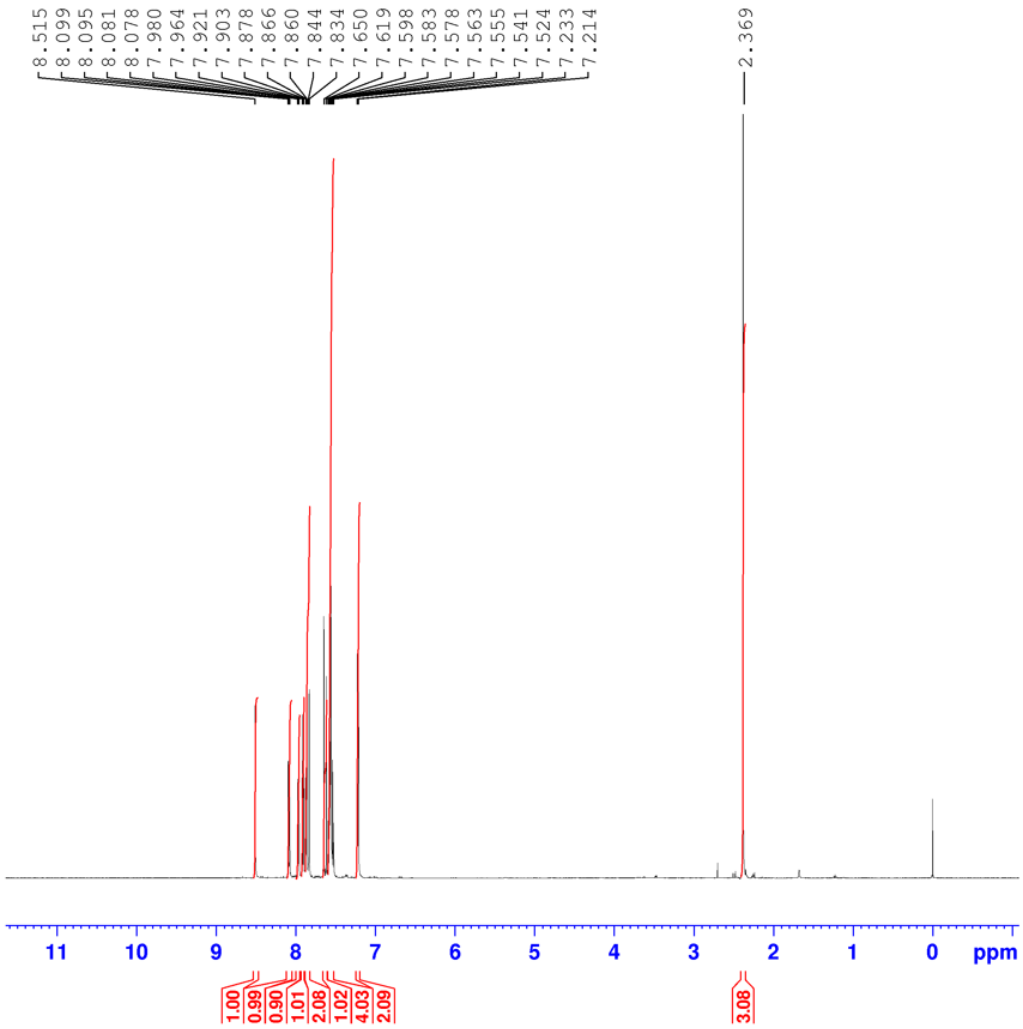

Figure S2: ^{13}C NMR Spectrum of compound **A**

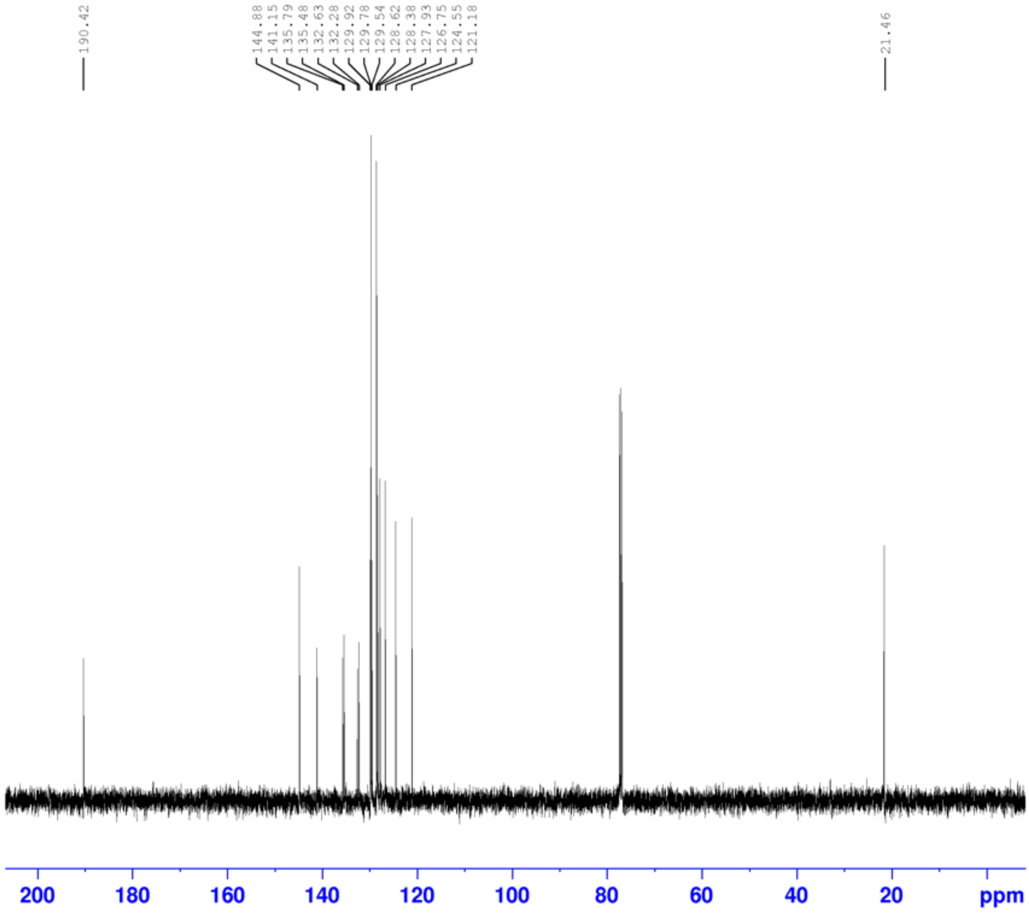

Figure S3: Mass Spectrum of compound **A**

Note : NBA
Inlet : Direct   Ion Mode : FAB+
RT : 1.38 min   Scan#: (5,8)
Elements : C 200/0, H 200/0, O 2/0
Mass Tolerance   : 20ppm, 10mmu if m/z < 500, 20mmu if m/z > 1000
Unsaturation (U.S.) : -0.5 - 100.0

Observed m/z	Int%	Err[ppm / mmu]	U.S.	Composition
272.1197	37.6	-1.6 / -0.4	13.0	C 20 H 16 O
273.1280	100.0	+0.1 / +0.0	12.5	C 20 H 17 O

[Theoretical Ion Distribution]   Page: 1
Molecular Formula : C20 H17 O
(m/z 273.1279, MW 273.3544, U.S. 12.5)
Base Peak : 273.1279, Averaged MW : 273.3556(a), 273.3565(w)

m/z	INT.	
273.1279	100.0000	**
274.1313	22.5378	*************
275.1345	2.6165	**
276.1376	0.2089	
277.1406	0.0127	
278.1436	0.0006	

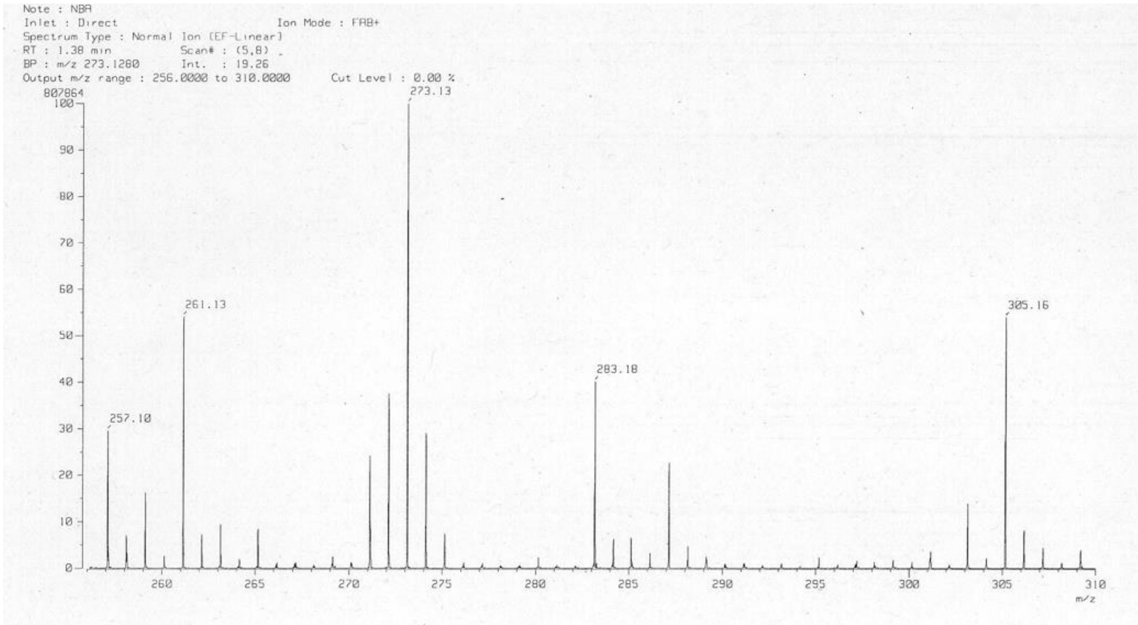

Figure S4: ^{1}H NMR Spectrum of compound **B**

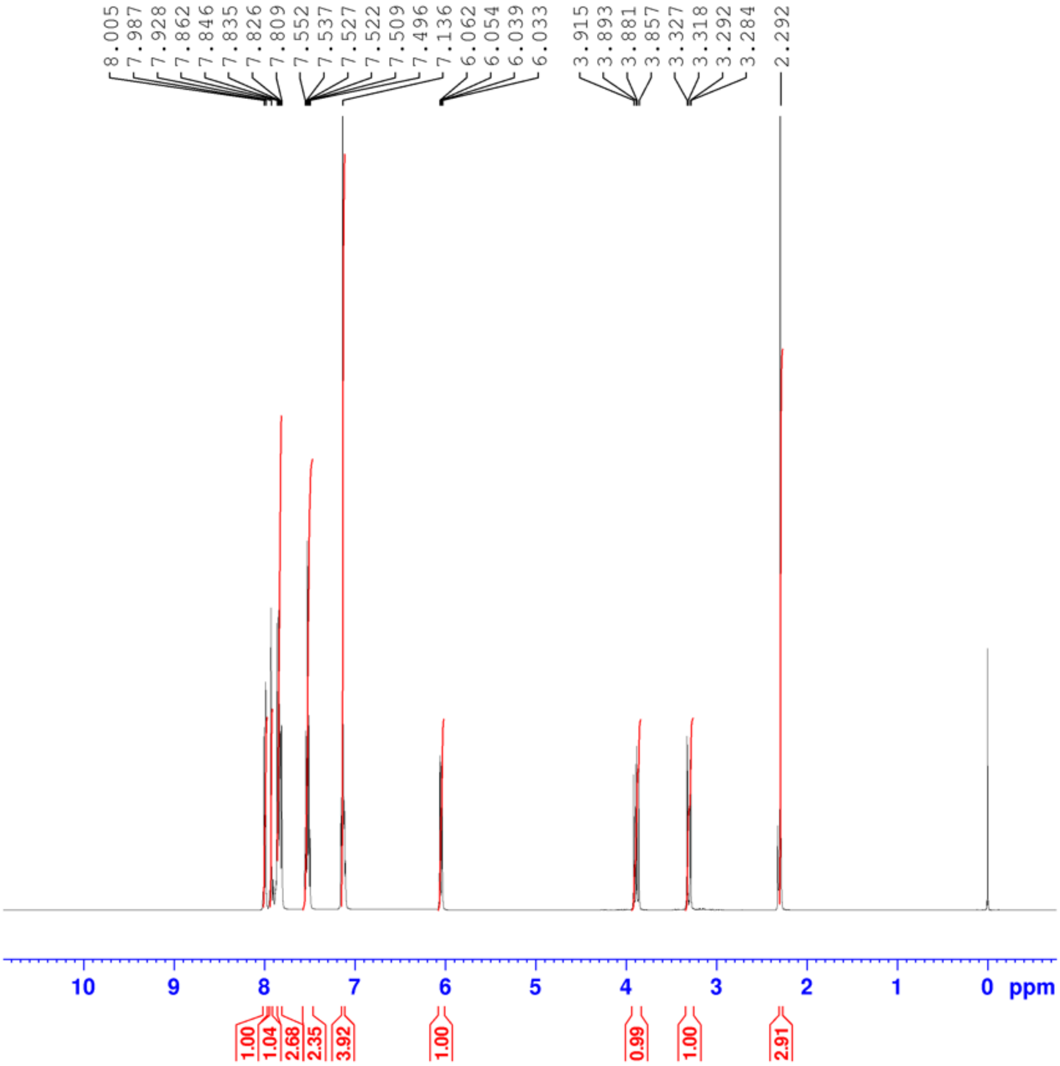

Figure S5: ^{13}C NMR Spectrum of compound **B**

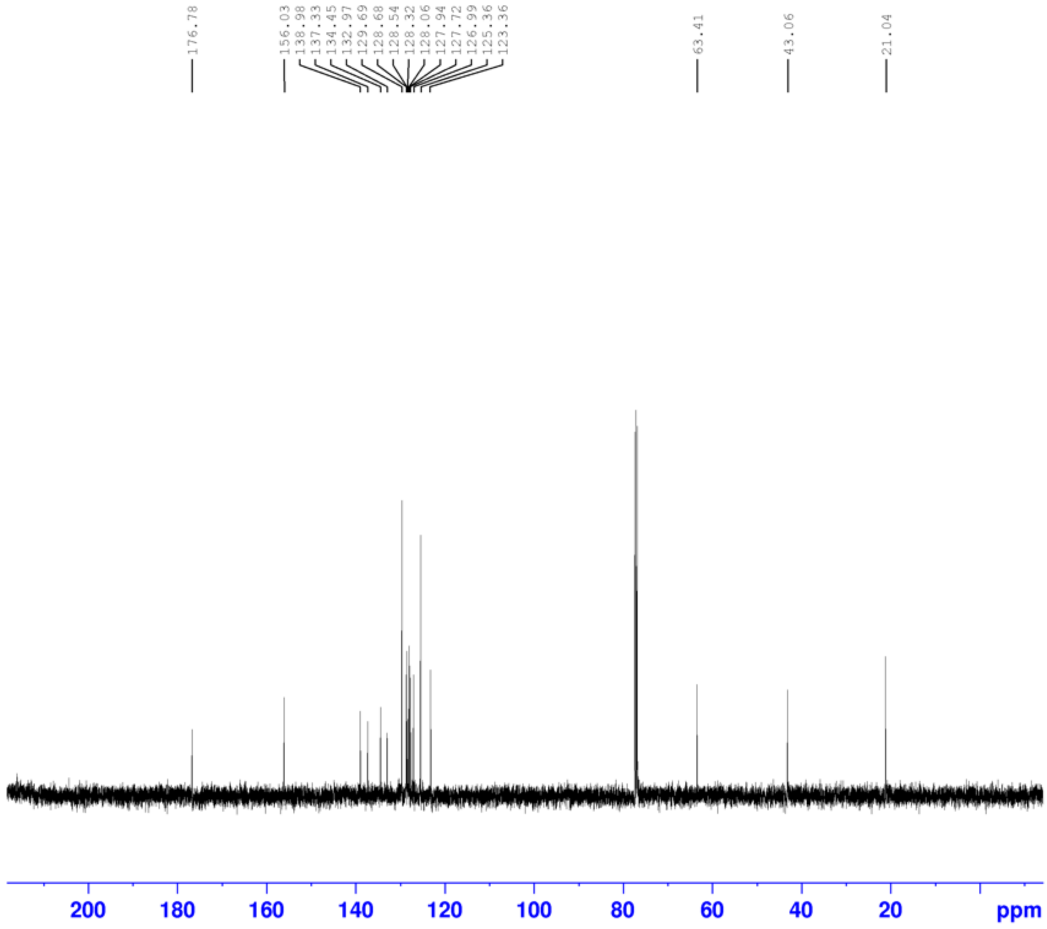

Figure S6: Mass Spectrum of compound **B**

```
Note : NBA
Inlet : Direct                                Ion Mode : FAB+
RT : 1.00 min                                 Scan#: (3,7)
Elements : C 200/0, H 200/0, N 4/2, S 2/0
Mass Tolerance        : 20ppm, 10mmu if m/z < 500, 20mmu if m/z > 1000
Unsaturation (U.S.) : -0.5 - 100.0

Observed m/z  Int%    Err[ppm / mmu]    U.S.  Composition
  346.1405   100.0     -18.8 /  -6.5    18.0  C 25 H 18 N 2
                       +17.6 /  +6.1    18.5  C 24 H 16 N 3
                       -28.5 /  -9.9    14.0  C 22 H 22 N 2 S
                        +7.8 /  +2.7    14.5  C 21 H 20 N 3 S
                        -1.9 /  -0.7    10.5  C 18 H 24 N 3 S 2
```

```
[ Theoretical Ion Distribution ]                                    Page: 1
Molecular Formula : C21 H20 N3 S
                         (m/z   346.1378, MW   346.4759, U.S. 14.5)
Base Peak :   346.1378, Averaged MW :   346.4759(a),   346.4770(w)

    m/z        INT.
  346.1378 100.0000 ***************************************************************
  347.1408  25.5483 ****************
  348.1378   7.5592 ****
  349.1385   1.3414 *
  350.1397   0.1647
  351.1409   0.0156
  352.1424   0.0012
```

```
Note : NBA
Inlet : Direct                          Ion Mode : FAB+
Spectrum Type : Normal Ion [EF-Linear]
RT : 1.00 min           Scan# : (3,7)
BP : m/z 346.1405       Int.   : 43.01
Output m/z range : 300.0000 to 354.0000      Cut Level : 0.00 %
```

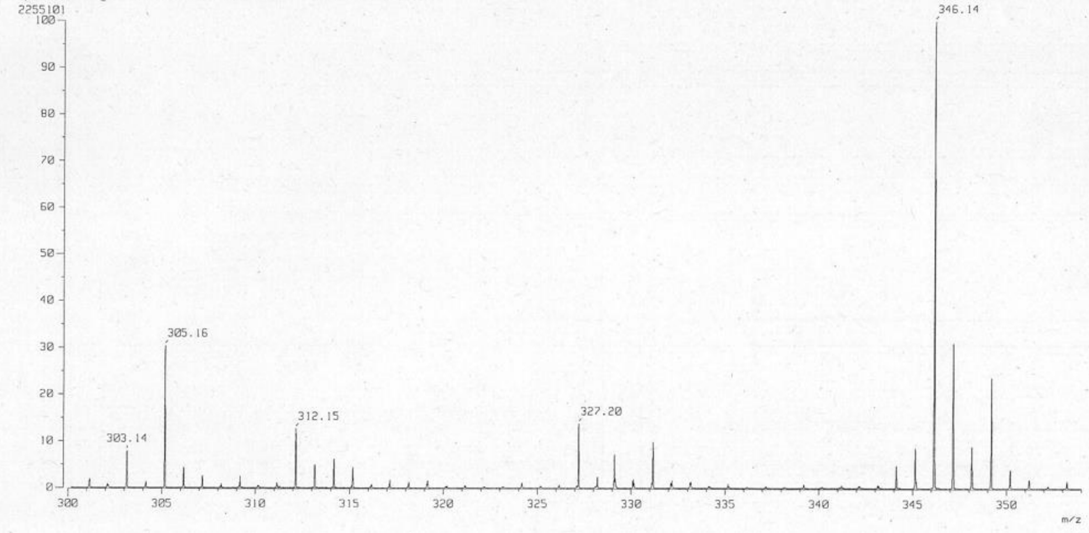

Supplement: Supplementary file 1 [file biomedicines-14-01651-s001.zip › biomedicines-4426228-supplementary.pdf]
